# Energy crisis and cartilage collapse: metabolic reprogramming of chondrocytes in osteoarthritis

**DOI:** 10.3389/fimmu.2026.1834080

**Published:** 2026-07-16

**Authors:** Fangzheng Yang, Qi Wang, Xuesai Zhu, Yizhi Yao, Tengbo Yu, Xiao Xiao

**Affiliations:** 1 Department of Orthopedic Surgery, Qingdao Municipal Hospital, Qingdao University, Qingdao, China; 2 Department of Orthopedic Surgery, Qingdao Municipal Hospital, Dalian Medical University, Qingdao, China; 3 Central Laboratories, Qingdao Municipal Hospital, University of Health and Rehabilitation Sciences, Qingdao, Shandong, China; 4 Department of Orthopedic Surgery, Key Laboratory of Orthopedics, Sports Medicine and Rehabilitation, Qingdao Municipal Hospital, University of Health and Rehabilitation Sciences, Qingdao, Shandong, China

**Keywords:** chondrocytes, energy metabolism, extracellular matrix, glycolysis, osteoarthritis

## Abstract

Osteoarthritis (OA), a degenerative joint disease imposing a significant global disease burden, exhibits pathological mechanisms far more complex than mere “cartilage wear”. Previous research has long centered on mechanical wear of articular cartilage and inflammatory responses. However, recent studies reveal that metabolic reprogramming and energy metabolism disorders constitute core metabolic alterations in OA. This review centers on the link between the energy crisis in articular chondrocytes and cartilage collapse. It systematically examines evidence suggesting that impaired oxidative phosphorylation (OXPHOS), increased glycolysis, and altered metabolic substrates may contribute to OA pathogenesis by modifying the local microenvironment and epigenetic regulation, thereby promoting inflammation and extracellular matrix (ECM) degradation. Furthermore, this review synthesizes recent research to propose novel therapeutic hypotheses, suggesting that OA is a highly heterogeneous disease at the metabolic reprogramming level, and often initiated or exacerbated by pathological biomechanical loads. Future efforts should reclassify OA based on metabolic reprogramming to develop targeted therapies addressing distinct cellular metabolism pathways—such as glycolysis inhibitors, mitochondrial protectants, and glutaminase inhibitors.

## 1 Introduction

OA is far from the traditional notion of “joint wear and tear”, but rather a complex disease characterized by metabolic heterogeneity and involving the entire joint. Its characteristic pain and functional impairment have become a leading cause of disability and loss of productivity worldwide. Between 1990 and 2019, the global burden of OA showed an overall upward trend, posing a significant public health challenge (1). It is reported that OA affects over 7% of the global population. The economic impact of OA is also substantial, with estimates indicating that the average annual loss per person due to OA ranges from US$700 to US$15,600 across different regions of the world (2). The current conservative treatment strategy, centered on symptom management, may provide short-term relief from pain but cannot reverse the disease progression. Ultimately, the majority of patients inevitably become reliant on joint replacement surgery. In recent years, the academic research perspective has shifted from the macro-level phenomenon of cartilage collapse to the underlying metabolic dysregulation driving this process. Among these factors, the energy crisis and metabolic reprogramming within chondrocytes have been implicated as critical contributors that may initiate and accelerate cartilage destruction (3, 4). Critically, abnormal biomechanical loading is not merely a consequence of joint dysfunction (5) but acts as a primary upstream trigger (6), converting of pathological mechanical stress into intracellular metabolic signals—a process known as mechanotransduction (7) — represents a key early event in OA (8, 9).

*In vitro* studies using primary bovine and human articular chondrocytes indicate that, chondrocytes primarily rely on glycolysis for energy production (10). Research indicates that this metabolic pattern adapts to the cartilage microenvironment, enabling glycolysis to supply sufficient energy for collagen and proteoglycan synthesis, thereby maintaining cartilage health (11). However, recent research indicates that during the progression of OA, this homeostasis is disrupted. Chondrocytes undergo metabolic reprogramming, characterized by impaired mitochondrial OXPHOS, increased glycolysis, and altered metabolic substrates (11–13); this shift is thought to trigger subsequent changes: insufficient ATP production via OXPHOS may lead to an “energy crisis”, which could cause chondrocytes to compensatorily increase glycolysis, producing excessive amounts of lactate (14), reactive oxygen species (ROS), and inflammatory factors. These changes may alter the local microenvironment, potentially promoting inflammatory responses and ECM degradation (11, 13, 15), and eventually lead to cartilage collapse.

Consequently, research into metabolic reprogramming and energy conversion in chondrocytes within hypoxic microenvironments has emerged as a critical field of study. This paper aims to systematically elucidate the metabolic patterns of normal cartilage and how metabolic reprogramming in chondrocytes during OA induces an “energy crisis” that triggers cartilage collapse. Furthermore, it explores potential targets and developmental prospects for future precision therapies for arthritis based on metabolic subtypes.

## 2 Healthy cartilage metabolism

Before delving into the intricate metabolic reprogramming within OA, it is essential to first revisit how healthy articular cartilage establishes and sustains its glycolysis-based metabolic pattern within its unique hypoxic microenvironment. This pattern not only serves as the energy source for chondrocytes to synthesize ECM and maintain tissue structure and function, but also constitutes a dynamic metabolic signaling network. The disruption of its homeostasis is considered an early and critical event in the pathological cascade.

### 2.1 Glucose metabolism

Glucose is the most commonly utilized metabolic substrate in most normal cells (16, 17). In normal cartilage, owing to the absence of blood vessels, oxygen is primarily derived from diffusion through synovial fluid and the subchondral bone vasculature (18, 19). Consequently, oxygen concentrations remain at merely 1%–6%, significantly lower than in normal tissues (20, 21). *In vitro* experiments using bovine articular chondrocytes demonstrate that under hypoxic conditions of 10% oxygen concentration (approximating the oxygen levels near cartilage surfaces), chondrocyte pellet volume and ECM mass reach their maximum levels. Furthermore, chondrocytes continue to exhibit vigorous glycolysis even under hyperoxic conditions (22). The underlying reason for its vigorous glycolysis lies in the metabolic reprogramming of chondrocytes during cartilage growth and development, induced by genes such as SOX9. This process enhances their adaptability to hypoxic conditions and boosts their capacity to synthesize ECM components, including proteoglycans (23, 24), This indicates that in healthy cartilage, chondrocytes primarily rely on glycolysis for energy production (10). Induction of glycolysis-related rate-limiting enzymes via HIF-1α or TGF-β1 increases glycolytic rate and reduces ROS production, thereby maintaining cartilage homeostasis (25).

### 2.2 Lipid metabolism

In addition to glucose, lipid metabolism is also increasingly attracting the attention of researchers. Chondrocytes regulate cartilage metabolism by expressing enzymes involved in lipid metabolism, while simultaneously being regulated by lipids circulating in the blood and those present within the cartilage matrix (26). In normal cartilage, three classes of lipids are typically present: fatty acids, phospholipids, and cholesterol, Among these, fatty acids may be categorized as saturated or unsaturated fatty acids. Saturated fatty acids may promote cartilage inflammation by regulating HIF-1α levels; n-6 unsaturated fatty acids may promote cartilage inflammation via the arachidonic acid-COX pathway (27, 28), n-3 polyunsaturated fatty acids can suppress cartilage inflammation by eliminating ROS, inhibiting neutrophil aggregation, reducing chemokine and cytokine mediators, and promoting tissue repair and regeneration (29), n-9 unsaturated fatty acids maintain cartilage health by inhibiting angiogenesis and other mechanisms (30, 31). Phospholipids can be classified into glycerophospholipids or sphingolipids. Glycerophospholipids are considered important biomarkers for OA (32, 33). Studies have shown that the lipid profile of synovial fluid in patients with OA reflects the severity of the condition; for example, patients with early-stage OA have higher levels of phosphatidylcholine in their synovial fluid (33). Sphingolipids, on the other hand, can promote chondrocyte proliferation (34). For instance, multiple *in vitro* studies have confirmed that sphingosine-1-phosphate (S1P) can activate downstream phospholipase C via G protein-coupled receptors, increase intracellular calcium ion concentrations, thereby activating ERK, and ultimately promote the proliferation of rat primary chondrocytes (35). Cholesterol synthesis may facilitate endochondral ossification by enhancing IHH gene expression (27).

### 2.3 Amino acid metabolism

Apart from carbohydrates and fatty acids, another major category of important metabolic substrates comprises amino acids. A substantial body of research currently focuses on the relationship between amino acid profiles and articular cartilage. Studies have shown that, compared to healthy cartilage, levels of several amino acids—including tryptophan, methionine, cysteine, aspartic acid, and asparagine—are reduced in osteoarthritic cartilage (28, 36). In addition, various diseases have been shown to be associated with changes in amino acid profiles (37). Studies have demonstrated that the amino acid profiles of synovial fluid differ in cases of ligament and meniscus injuries (38); following ankle fractures, levels of 19 amino acid metabolites are significantly elevated in patients’ synovial fluid (39); and the specific amino acid biomarker o-tyrosine has been identified in the synovial fluid of patients with rheumatoid arthritis (40).

In summary, within the hypoxic microenvironment of normal cartilage, chondrocytes maintain a metabolic equilibrium for the three primary nutrients: carbohydrates, fatty acids, and amino acids. Energy production and consumption are broadly balanced, with no energy crisis present.

## 3 Cartilage metabolism in OA

In OA, the metabolic balance mentioned earlier is profoundly altered (41). Metabolic reprogramming in chondrocytes is proposed to trigger an energy crisis, which may activate inflammatory signaling pathways, and promote ECM degradation. Consequently, the following discussion will focus on the specific mechanisms and key nodes of metabolic reprogramming in OA. This examination aims not only to elucidate metabolic alterations within the pathological state but also to reveal their relationship with inflammatory responses and ECM degradation.

### 3.1 Mitochondrial abnormalities

Compared to normal chondrocytes, chondrocytes in OA exhibit abnormalities in mitochondrial signaling molecules and mitochondrial membranes, as well as excessive production of reactive oxygen species. This leads to a reduction in energy production via OXPHOS within the mitochondria, creating a crisis of energy supply and demand imbalance, forcing the cells to rely on glycolysis to compensate for the energy deficit.

#### 3.1.1 Abnormalities in signaling molecules

Research into the mechanisms underlying reduced mitochondrial energy production in osteoarthritic chondrocytes has primarily focused on mitochondrial clearance impairment (42–45).

Sirtuins (SIRT), a class of proteins closely associated with cellular metabolism, growth, and aging, are increasingly recognized as important regulators of mitochondrial clearance in chondrocyte senescence during OA (42, 43, 46–48). Their signaling pathways are intrinsically linked to this process, with seven distinct subtypes currently identified. *In vitro* studies have reported that evodiamine can attenuate IL-1β-induced chondrocyte damage, an effect linked to activation of the SIRT1/PGC-1α pathway, increasing mitochondrial DNA copy number, promoting mitochondrial biosynthesis, reducing ROS production, and improving mitochondrial function (46). In contrast, homocysteine exacerbates mitochondrial dysfunction, oxidative stress, and chondrocyte damage by inhibiting the SIRT1/AMPK/PGC-1α pathway (47), consistent with the clinical association that hyperhomocysteinemia may promote the progression of OA (49).

However, the role of SIRT1 in OA exhibits a certain degree of duality. On the one hand, moderate activation of the SIRT1/PGC-1α pathway enhances mitochondrial biogenesis, maintains the homeostasis of the chondral ECM, and suppresses the expression of catabolic enzymes such as matrix metalloproteinase 13 (MMP-13), thereby exerting a protective effect (50, 51). On the other hand, some studies indicate that excessive or sustained SIRT1 activation may deplete mitochondrial NAD^+^, leading to NAD^+^ pool depletion, reduced ATP production, and increased MMP-13 expression and ECM degradation, ultimately triggering an energy crisis and enhanced catabolism (52, 53). These discrepancies likely reflect context-dependent effects, which may be influenced by factors such as the disease stage (54), the choice of *in vitro* vs. *in vivo* models (51, 55–59), and the concentration or specificity of SIRT1 modulators employed (60–62).Future research should employ standardized models and approaches that combine NAD^+^ supplementation with SIRT1-modulating strategies to elucidate the mechanisms underlying the role of SIRT1 in OA.

Beyond SIRT1, other sirtuins also play crucial roles in chondrocyte biology (63). For example, both SIRT2 and SIRT3 are involved in mitochondrial quality control and the regulation of inflammatory responses: SIRT2 deacetylates NF-κB p65 to inhibit NLRP3 inflammasome activation (48, 64), while SIRT3, through axes like SIRT3-PINK1-PKM2, promotes mitochondrial renewal, thereby reducing ROS and preventing metabolic reprogramming (42, 65–68). SIRT4 and SIRT5 are implicated in metabolic stress responses; SIRT4 deficiency impairs mitophagy (43, 69), and SIRT5 inhibits ferroptosis (70). Finally, dysregulation of SIRT6 and SIRT7 compromises mitochondrial integrity (71) and autophagy (72, 73), respectively, further contributing to OA pathogenesis. This indicates that the Sirt family plays a crucial role in metabolic crises in chondrocytes.

To provide a concise overview of the functions of the SIRT family proteins mentioned above, we have compiled **Table 1**, which lists the SIRT family proteins currently known to play a role in OA, along with their regulatory pathways and therapeutic agents.

**Table 1:** SIRT family proteins involved in OA: their functions, key effects, and therapeutic agents.

Sirtuin	Change/function in OA	Key downstream effects / mechanisms	Potential pharmacological interventions	References
SIRT1	Dual role: moderate activation protects cartilage, but excessive activation depletes NAD^+^ and promotes catabolism	Promotes mitochondrial biogenesis → maintains ECM; inhibits MMP-13. Overactivation reduces ATP and increases MMP-13	Evodiamine	(46, 47, 49–53)
SIRT2	Decreased expression may enhance inflammation	Deacetylates NF-κB p65 → inhibits NLRP3 inflammasome → reduces pyroptosis	Celastrol (upregulates SIRT2)	(48, 64)
SIRT3	Reduced in OA chondrocytes; restoration preserves mitochondrial function	Deacetylates PINK1 and PKM2 → promotes mitochondrial renewal and mitophagy; reduces ROS	Asperosaponin VI, polydatin, chondrocyte-mimetic nanoplatform	(42, 65–68)
SIRT4	Downregulated in OA; deficiency drives senescence	Loss of SIRT4 impairs mitophagy → mitochondrial dysfunction and chondrocyte senescence	–	(43, 69)
SIRT5	Chondroprotective	Desuccinylates ACSL4 → inhibits ferroptosis	Curcumin (acts partly through SIRT5)	(70)
SIRT6	Downregulation compromises mitochondrial integrity	Maintains mitochondrial function, reduces oxidative stress; deficiency promotes senescence	–	(71)
SIRT7	Dysregulation leads to impaired autophagy	Activates autophagy → protects cartilage; deficiency accelerates OA	–	(72, 73)

This table illustrates the role of the SIRT family in OA, as well as its downstream effects and currently known drugs that may influence it.

In mechanically induced OA models, PINK1 suppresses chondrocyte senescence and inhibits OA progression by promoting the clearance of senescent and defective mitochondria through inhibition of the p38 MAPK/NF-κB pathway (42, 44), while ginsenosides or curcumin may alleviate the progression of OA by mediating mitochondrial phagocytosis through the AMPK-PINK1-Parkin pathway (74, 75). Acetylcarnitine or adipose-derived mesenchymal stem cell-secreted exosomes can enhance PINK1/Parkin-mediated mitochondrial engulfment, thereby promoting the clearance of damaged mitochondria. This reduces ROS production and suppresses inflammatory responses (74, 76).

#### 3.1.2 Mitochondrial membranes, ion concentration and structural abnormalities

Currently, research focusing on mitochondrial membrane potential and structure remains limited, with the majority of studies centering on mitochondrial calcium overload-induced chondrocyte apoptosis. Research indicates that the calcium channel CaV3.3 regulates the coupling of mitochondrial calcium redox through interaction with the mitochondrial calcium uncoupler (MCU) complex. Diminished CaV3.3 expression disrupts mitochondrial ultrastructure, accompanied by MICU1 downregulation and loss of MCU gating fidelity. This permits pathological calcium influx into mitochondria, triggering excessive ROS production and bioenergetic collapse, thereby activating apoptosis (77), In the temporomandibular joint osteoarthritis (TMJOA) model, Optin, a vision-related protein, regulates the IP3R-GRP75-VDAC1 complex by interacting with glycogen synthase kinase 3β (GSK3β), thereby reducing mitochondrial calcium concentrations and inhibiting chondrocyte apoptosis (78). The mitochondrial-associated peptide MOTS-c improves mitochondrial structure and membrane potential in chondrocytes via the Nrf2/TXNIP/NLRP3 axis during LPS-induced oxidative stress (79).

#### 3.1.3 Mitochondria generate large quantities of ROS

ROS are unavoidable by-products of mitochondrial OXPHOS, and their production increases in aging or structurally damaged mitochondria. Research evidence indicates that mitochondrial ROS generation—including superoxide anion, hydrogen peroxide, and nitric oxide—plays a pivotal role in the pathogenesis of OA (80, 81).

The superoxide anion within mitochondria is primarily generated by errors in electron transfer within the mitochondrial electron transport chain during OXPHOS (81) and by non-mitochondrial membrane-bound NADPH oxidases. Consequently, ROS production is frequently accompanied by a decline in mitochondrial membrane potential, reduced ATP production, and diminished mitochondrial DNA. Peroxide anions can directly oxidize proteins, thereby disrupting the mitochondrial membrane and significantly altering signal transduction, gene expression, and the cell cycle. Peroxide anions can be acted upon by SOD enzymes and converted into H_2_O_2_, while catalase and peroxidases catalyze the reduction of H_2_O_2_ to water, thereby exerting a protective effect (82).

Literature indicates that OA induced by multiple factors can lead to increased production of ROS within chondrocyte mitochondria, thereby causing cellular damage and MMP13-mediated degradation of the ECM (80, 83, 84). Studies have reported that IL-1β-stimulated OA chondrocytes exhibit elevated ROS generation, mitochondrial membrane damage, accumulation of damaged mitochondria, and increased apoptosis rates (85). Sodium iodoacetate (MIA), commonly employed for modeling OA in animals, increases ROS production and reduces mitochondrial membrane potential in chondrocytes. The antioxidant N-acetylcysteine fully protected cells from MIA-induced apoptosis by reducing ROS production, increasing mitochondrial membrane potential, and inhibiting cytochrome c and caspase-3, indicating that ROS generation plays a critical role in MIA-induced chondrocyte apoptosis (86). Currently, multiple approaches have been developed to mitigate ROS-induced damage across various stages of mitochondrial ROS production, with the majority targeting Parkin. Literature reports indicate that supplementation with PDZK1 or the ROS scavenger Mitoquinone alleviates chondrocyte senescence and cartilage degeneration (87). Mesenchymal stem cells can transfer mitochondria to co-cultured chondrocytes, thereby reducing ROS levels, conferring antioxidant stress resistance, and enhancing anti-apoptotic capacity (88), while Parkin translocates to depolarized/damaged mitochondria and recruits p62/SQSTM1, thereby clearing damaged mitochondria, reducing ROS levels, and inhibiting chondrocyte apoptosis (85). The use of antioxidants, such as hollow Prussian blue nanoenzyme particles encapsulated in TPP-modified membranes, can precisely eliminate ROS within mitochondria. This approach reduces mitochondrial DNA leakage, inhibits the cGAS-STING-NF-κB signaling pathway, enhances chondrocyte function, and suppresses MMP activity while inhibiting ECM degradation (89). Other antioxidants, such as Mn_3_O_4_/UIO-TPP nanozymes (90) and zero-valent selenium-enriched hydrogen-releasing nanozymes (91), can also protect chondrocytes by scavenging mitochondrial ROS. Tiopronin targets and activates the Bnip3-PINK1-Parkin signaling pathway, thereby promoting ECM synthesis by enhancing the clearance of senescent mitochondria and reducing ROS, thus alleviating cartilage degradation (45). Yanghe decoction and protocatechuic aldehyde can eliminate damaged mitochondria by regulating the AMPK-SIRT3 pathway and activating PINK1/Parkin (92, 93), while Gel-loaded catalase can reduce ROS level by activating the SIRT3-UPR pathway, thereby promoting the clearance of senescent and damaged mitochondria to improve OA (94).

### 3.2 Enhanced glycolysis

In OA, impaired mitochondrial OXPHOS leads to an energy deficit, causing chondrocytes - which already rely on glycolysis (12, 95) - to undergo pathological overactivation of glycolysis to compensate for the energy shortfall, fueling a vicious cycle of inflammation and matrix destruction.

#### 3.2.1 Increased glucose transport

Glucose transporters (GLUT) are a family of membrane proteins responsible for transporting glucose. Under physiological conditions, a high-glucose environment negatively regulates GLUT-1 expression and glucose uptake in normal chondrocytes; however, chondrocytes in OA have lost this downregulation mechanism. This persistent dysregulation of glucose transport under hyperglycemic conditions leads to intracellular glucose accumulation, inducing the massive production of ROS and advanced glycation end products (AGEs), thereby accelerating the progression of OA (96, 97). Clinically, PET-CT scans have confirmed significantly increased glucose uptake at lesion sites in OA patients (98); *in vitro* studies further indicate that mechanical loading, inflammatory factors (such as IL-1β), and glucosamine can all influence chondrocyte function by regulating GLUT activity (99). In summary, dysregulation of GLUT-mediated glucose uptake is a key pathogenic mechanism driving the onset and progression of OA.

#### 3.2.2 Increased expression of the rate-limiting enzyme for cytoplasmic glycolysis

In addition to increased glucose uptake, mechanical forces and other pathogenic factors trigger intracellular signaling cascades through pathways such as PIEZO1, thereby upregulating the expression and activity of key glycolytic enzymes such as HK II (100), thus may lead to increased glycolytic flux (101–103), which is also a key pathogenic mechanism of OA.

Non-targeted metabolomic analysis of a chicken OA model revealed decreased levels of glycolytic intermediates (fructose-6-phosphate, fructose-1,6-diphosphate, and 2-phosphoglycerate) in cartilage tissue, while levels of the end product, pyruvate, were elevated; concurrently, LDHA gene expression was upregulated and the rate of extracellular acidification increased (102). The overall metabolic profile of OA chondrocytes was characterized by a marked increase in glycolytic activity, accompanied by impaired OXPHOS. This enhanced glycolysis was also confirmed in *in vitro* experiments using chondrocytes from patients with knee OA (103).

Hexokinase (HK) can be divided into two subtypes: HK I and HK II. Among these, HK II is the key enzyme facilitating the flow of glucose toward glycolysis (104). Research has suggested that mechanical loading may promote OA through the PIEZO1-mesenchymal stem cell-HK II-macrophage migration inhibitory factor (MIF)-macrophage axis (100); research has also demonstrated that within osteoarthritic chondrocytes, the pro-inflammatory cytokine TGF-β1 promotes the expression of HK II and GLUT-1, inducing increased glycolysis and reduced OXPHOS. Furthermore, overexpression of HK II elevates the RNA expression levels of pro-inflammatory cytokines such as TNFα, IL-6, IL-8, and MMPs (25). A clinical study focusing on HK II has also confirmed that the expression of HK II in peripheral blood mononuclear cells is increased in both rheumatoid arthritis (RA) and OA cohorts (105). Research has demonstrated that HK II levels in osteoarthritic chondrocytes are regulated by pathways including the PI3K/Akt pathway, the NF-κB pathway, and the NLRP3 inflammasome pathway (106). These findings demonstrate that investigating the role of HK II in the pathogenesis of OA holds significant importance for identifying metabolic targets in OA treatment.

Traditional views hold that 6-phosphofructo-2-kinase/fructose-2,6-biphosphatase 3 (PFKFB3) is a key enzyme in glycolytic metabolism and a significant accelerator of glycolytic metabolic rates (107), it can enhance glycolytic activity by synthesizing 2,6-diphosphate fructose, thereby activating phosphofructokinase (PFK), the second rate-limiting enzyme of glycolysis. In OA, the PFKFB3 gene exhibits abnormal methylation (108), and lactic acid-induced H4K12 lactylation promotes the transcription of pro-inflammatory genes, thereby reinforcing the PFKFB3-mediated positive feedback loop, which further amplifies glycolysis and exacerbates chondral inflammation (109). Certain drugs, such as Songorine, can improve OA by binding to and inhibiting PFKFB3 (13), however, some studies indicate that PFKFB3 is suppressed in patients with OA (110). The complex role of PFKFB3 in OA may stem from multiple contributing factors. The conflicting findings regarding PFKFB3 in OA may result from disease-stage-dependent effects, model differences, and methodological variability. During early OA, PFKFB3 can amplify catabolism through a lactate-driven glycolytic-inflammatory positive feedback loop (13, 111), whereas in late-stage disease it appears to serve a protective role by counteracting endoplasmic reticulum stress and DNA damage (110, 111). Surgically induced models often show reduced PFKFB3 expression consistent with protection (111), while chemically induced models more readily reveal its pro-inflammatory functions (13). Moreover, because systemic PFKFB3 knockout is embryonically lethal (112), most studies employ partial knockdown or chemical inhibition; variations in the degree of inhibition achieved by these approaches may further contribute to contradictory outcomes. Standardized models and stage-specific analyses are therefore needed to define the precise function of PFKFB3 in OA.

The third rate-limiting enzyme in the glycolytic pathway is pyruvate kinase (PK), which comprises four distinct subunits: M1, M2, L1, and L2. Current research on PK primarily focuses on PKM2. Studies have shown that PKM2 normally exists as a tetramer, whereas elevated PKM2 expression and dimerization have been observed in osteoarthritic cartilage from both humans and mice (113). Specific knockout of PKM2 in chondrocytes or treatment with a PKM2 tetramer stabilizer can maintain mitochondrial function by promoting mitochondrial fusion and disrupting the interaction between PKM2 and ERK; this leads to ERK-dependent upregulation of mitochondrial protein 1 expression, thereby protecting cartilage tissue affected by OA (42, 114). In a mouse model of TMJOA, LincRNA-EPS has been shown to bind to serine/arginine-rich splicing factor 3 and inhibit PKM2 formation, thereby suppressing the PKM2/NF-κB pathway and inflammatory mediators (115).

#### 3.2.3 Increased lactate production within chondrocytes

As the primary metabolite of glycolysis, lactate plays a significant role in the pathophysiology of OA. However, a comprehensive understanding of how lactate influences cartilage metabolism remains elusive.

Within chondrocytes, lactate production is primarily governed by lactate dehydrogenase (LDHA), which occupies a pivotal position in pyruvate metabolism. Inhibition of LDHA suppresses glycolysis while promoting OXPHOS. Lactate produced by increased glycolysis can influence chondrocyte function through multiple pathways, including signaling transduction, epigenetic regulation, modulation of cellular energy and metabolic homeostasis, regulation of immunomodulation and inflammatory responses, direct contribution to an acidic micro-environment (116).

Researchers have measured lactate concentrations in synovial fluid, revealing significantly elevated levels in patients with OA (117, 118). This finding aligns with observations in IL-1β-treated inflammatory chondrocytes. Lactate inhibits enzyme activity by binding to UDP-glucose dehydrogenase, thereby reducing glycosaminoglycan synthesis. It further promotes inflammatory progression via the STAT1-MAPK signaling pathway or by binding to histone H3 (11, 116). During inflammatory states, lactic acid can increase ROS in chondrocytes, reduce M1 macrophage activation, and promote M2 macrophage polarization (104).

To provide a visual summary of the key enzymes involved in the processes of glycolysis and lactate production mentioned above, we have included **Table 2**, which illustrates several key enzymes along with their upstream and downstream pathways and specific drugs.

**Table 2:** Changes in the rate-limiting enzymes of glycolysis and its mechanism in OA.

Enzyme	Expression/Changes in OA	Upstream-regulators/Pathways	Impact on inflammation/ECM degradation	Drug/Intervention	References
HK II	Up-regulated	Positive regulator: TGF-β1, HIF-1α, mechanical stress/PIEZO1, PI3K/Akt pathway, NF-κB pathway, NLRP3 inflammasome	Increases the level of inflammatory factors and MMPs; promotes M1 macrophage activation and ECM degradation; and it enhances glycolysis	2-Deoxyglucose, Chrysin	(25, 100, 106)
		Negative regulator: Normal chondrocytes can downregulate GLUT-1/HK II under high-glucose conditions, but this feedback mechanism is lost in OA			
PFKFB3	Controversial: Some studies suggest an increase, while others suggest a decrease	Positive regulator: Lactate/H3K18la/H4K12la positive feedback loop; PI3K/Akt/CHOP pathway	Dual roles: Protective effects: Overexpression of PFKFB3 alleviates stress and inflammation, promotes the expression of aggrecan and type II collagen; Destructive effects: PFKFB3 participates in a lactate-mediated positive feedback loop, exacerbating cartilage inflammation	Songorine; Tiopronin	(13, 108–111)
		Negative regulator: TNF-α and IL-1β can suppress PFKFB3 expression; promoter methylation can silence PFKFB3 expression			
PKM2	Up-regulated, accompanied by the conversion of tetramers to dimers	Positive regulator: NF-κB pathway, ERK signaling, HIF-1α	PKM2 binds to NF-κB, leading to the release of inflammatory factors and MMPs; the PKM2-ERK interaction disrupts mitochondrial homeostasis	PKM2 Tetramer Stabilizer TEPP-46	(42, 113–115)
		Negative regulator: The SIRT3-PINK1 axis promotes PKM2 tetramerization; LincRNA-EPS binds to SRSF3 to inhibit PKM2 alternative splicing			
LDHA	Up-regulated	Positive regulator: HIF-1α, c-MYC, the stearoyl/lactate-HIF-1α axis, STAT1-MAPK signaling; the lactate-H3K18 lactylation-LDHA axis	Inhibits glycosaminoglycan synthesis; elevates ROS levels, leading to chondrocyte damage; promotes TPI1 gene transcription, creating a positive feedback loop of inflammation; accelerates ECM degradation	Oxamate; α-solanine; DCA	(11, 15, 101, 119–121)

This table lists the key enzymes involved in glycolysis and lactate production as reported in the literature, along with their upstream regulatory pathways and downstream metabolic effects.

### 3.3 Altered metabolic substrates—increased amino acid metabolism

In addition to the aforementioned increase in glycolysis and mitochondrial damage, the cellular energy crisis in OA is characterized by heightened amino acid metabolism, notably represented by increased glutamine catabolism.

Glutamine, a common intracellular amino acid, undergoes hydrolysis into ammonium ions and glutamic acid. The latter is subsequently converted into α-ketoglutarate (α-KG), which then enters the tricarboxylic acid cycle. This process generates ATP via the production of NADH and FADH2 (122). Moreover, within chondrocytes, glutamine is utilized in the assembly of collagen and glycosaminoglycans (123, 124). As one of glutamine’s metabolic products is the antioxidant GSH, glutamine possesses antioxidant properties.

Current research has focused on glutamine metabolism in chondrocytes during the progression of OA. Studies indicate that synovial fluid levels of glutamate and glutaminase (GLS) are significantly elevated in OA patients, while glutamine and α-KG levels are comparatively low (125). In patients with TMJOA, intra-articular hyaluronic acid injections led to improvements in VAS pain scores and mouth opening range, as well as a significant decrease in glutamate levels, suggesting that glutamate degradation may increase during the progression of OA (126). The expression of glutamine metabolism-related proteins c-MYC and GLS1 increased in OA but decreased following exosome therapy (127). Animal studies have demonstrated that L-glutamine injections are comparable to glucosamine sulfate and celecoxib in improving the pathological progression and clinical efficacy of OA (128), this therapeutic effect may be achieved by enhancing GFPT1, thereby increasing collagen II synthesis and reducing MMP13 expression (129), some studies also suggest that this therapeutic effect is mediated through the JNK and NF-κB pathways or the TGF-β1-SMAD2/3-lncRNA-NKILA pathway (130–132). Inhibition of glutamyl transpeptidase can mitigate IL-1β and MIA-induced OA and ECM degradation via the NF-κB pathway (133).

**Figure 1:**
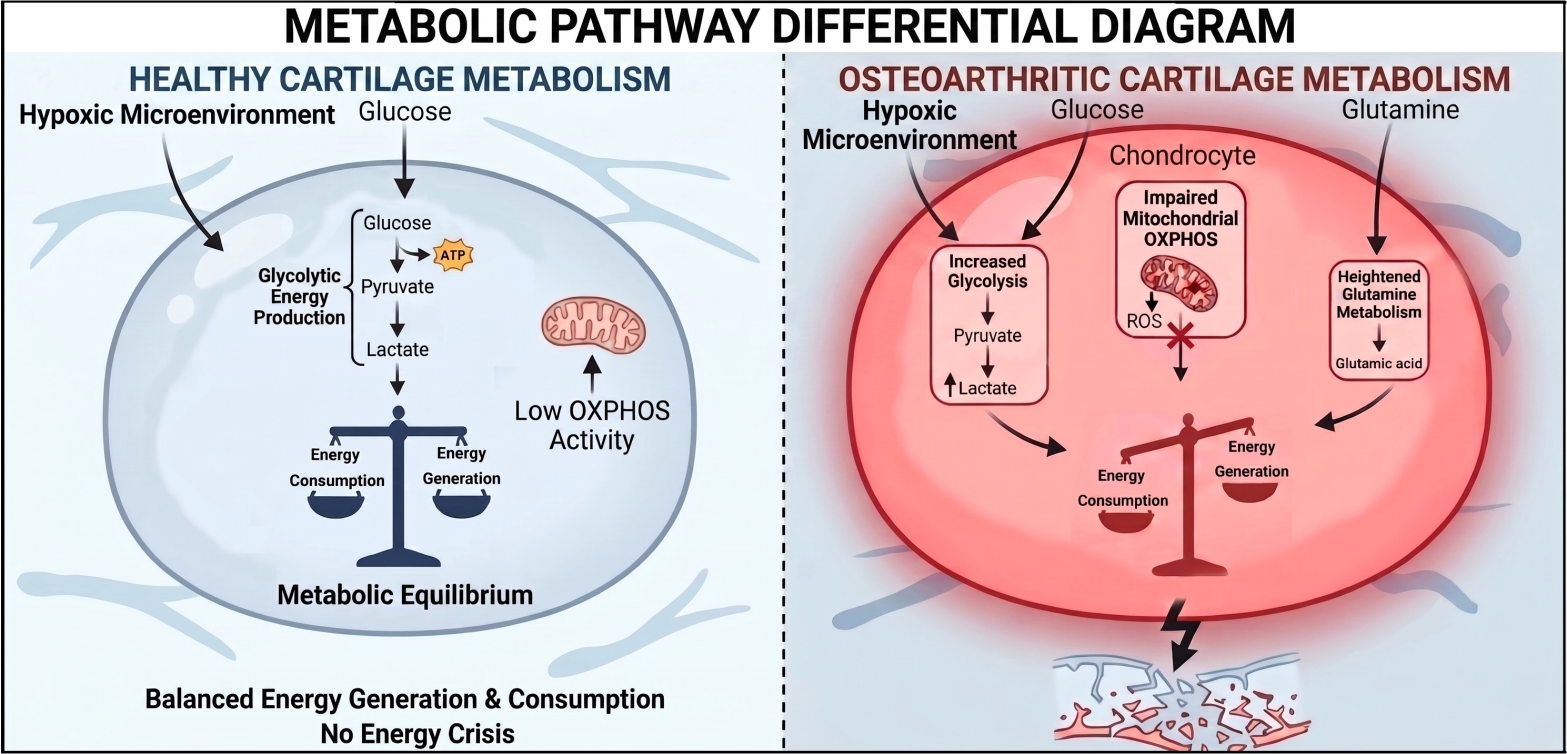
Metabolic pathway differential diagram. This image illustrates the mechanisms of metabolic reprogramming and energy crisis in normal and OA chondrocytes: in healthy cartilage, energy production primarily relies on glycolysis, with minimal contribution from OXPHOS; whereas in OA chondrocytes, lactate production from glycolysis increases, OXPHOS is impaired and generates substantial ROS, while glutamine metabolism also intensifies.

In summary, the key features of chondrocyte metabolic reprogramming in OA are enhanced glycolysis, mitochondrial abnormalities, and increased glutamine catabolism (**Figure 1**). These primarily lead to two major outcomes: inflammatory damage to chondrocytes and degradation of the extracellular matrix. These will be outlined separately below.

## 4 Mechanisms of inflammatory damage to chondrocytes

The aforementioned metabolic reprogramming establishes the energy and material basis for cartilage collapse. However, its pathological significance extends far beyond this: metabolic byproducts can drive inflammation by altering the local microenvironment and epigenetic regulation. The formation of this “metabolite-inflammation” axis signifies that metabolic reprogramming and the inflammatory cascade have entered a phase of positive feedback, thereby rapidly precipitating cartilage collapse. A substantial body of research is currently focused on this inflammatory mechanism.

### 4.1 Metabolic by-products alter the local microenvironment, leading to inflammation

As previously discussed, during the pathogenesis of OA, multiple mechanisms lead to increased glycolysis in chondrocytes, resulting in the release of substantial amounts of lactic acid into the local microenvironment. This lactic acid, on the one hand, causes acidification of the local microenvironment, with a persistent decrease in extracellular pH within the cartilage, meniscus, and osteochondral interface (134, 135). On the other hand, it induces extensive protein lactylation (Kla), activating inflammatory pathways in chondrocytes and triggering the release of inflammatory mediators, thereby contributing to the development of OA (41).

Literature indicates that as a metabolic byproduct, lactic acid simultaneously elevates intracellular NADPH levels while also activating the HCAR1-PI3K/Akt signaling pathway to upregulate NOX4. This promotes ROS production and chondrocyte damage, concurrently enhancing the expression of inflammatory cytokines IL-6 and CCL3/4 (119). Furthermore, elevated lactate levels in the microenvironment activate the UGDH-STAT1-MAP3K8 signaling pathway (11), potentially triggering downstream inflammatory pathways such as NF-κB. Utilizing stearic acid activates the LDHA-α-lactic acid-HIF-1α axis in chondrocytes, thereby increasing production of inflammatory cytokines including IL-6, TNF-α, and IL-1β (136). In a mouse model of TMJOA, inhibition of the SRSF2/PKM2/NF-κB pathway via linc-RNA suppresses lactate production by inhibiting the key rate-limiting enzyme of glycolysis, thereby reducing inflammatory cytokine levels (115).

### 4.2 Metabolites influence epigenetic regulation

Epigenetic regulation, such as histone modifications, DNA methylation, RNA methylation, and non-coding RNAs, also plays a significant role in the pathogenesis of OA and mediates the therapeutic effects of numerous promising therapeutic agents (137), while metabolic by-products such as lactic acid can alter epigenetic states through processes like Kla, thereby mediating metabolic reprogramming in chondrocytes.

Previously, scholars discovered a novel form of protein modification termed Kla (138), while lactic acid is the key precursor for histone Kla (139). Research indicates that in LPS-induced osteoarthritic chondrocytes, a positive feedback loop forms involving increased lactate dehydrogenase activity – elevated lactate levels – heightened H3K18la – enhanced TPI1 transcriptional activity – intensified glycolysis, thereby leading to chondrocyte damage and fibrosis (15). In osteoarthritic chondrocytes, demethylation of the MMP-3 gene promoter has been observed to facilitate binding of transcription activator 4 to the promoter, which may activate MMP-3 expression and promote ECM degradation (140). Previously, scholars induced inflammatory responses in the ATDC5 cell line via IL-1β, observing an upward trend in both RNA methyltransferase 3 expression levels and overall intracellular RNA m6A levels, alongside activation of the NF-κB signaling pathway. Conversely, silencing RNA methyltransferase 3 in a mouse model of OA reduced MMP-13 expression while promoting collagen II expression, thereby inhibiting ECM degradation (141). Moreover, in osteoarthritic chondrocytes, elevated levels of the lncRNA PILA bind to protein arginine methyltransferase 1 and enhance its activity, which is associated with increased NF-κB signaling; this mechanism has been proposed to contribute to OA pathogenesis (142).

In summary, as outlined above, the multifactorial and matrix-mediated “metabolite-inflammation” axis leads to inflammatory damage in chondrocytes.

## 5 Vigorous ECM degradation

Based on the above discussion of the energy crisis and metabolic reprogramming, a new question emerges: how does the energy crisis observed in OA drive ECM degradation, leading to cartilage collapse?

The ECM of articular cartilage comprises proteoglycans and embedded collagen and elastic fibers. Under normal conditions, a dynamic equilibrium is maintained through the anabolic synthesis by chondrocytes and the catabolic degradation mediated by MMPs/cysteine cathepsins. However, in OA, chondrocyte anabolism diminishes while catabolism increases, resulting in structural and functional disruption of the ECM.

### 5.1 Alterations in the local microenvironment lead to ECM degradation

Research indicates that increased glycolysis in chondrocytes may elevate local lactic acid levels, which in turn could upregulate MMP-3/13 and ADAMTS-4 expression and impair type II collagen and glycosaminoglycan synthesis, processes believed to contribute to cartilage degradation (11, 119). Sodium dichloroacetate or 2-deoxyglucose can inhibit glycolysis, thereby reducing lactate production and suppressing MMP3, MMP13 and ADAMTS4 (103), increasing the expression of collagen II and proteoglycan (11). Moreover, cysteine cathepsins responsible for ECM remodeling exhibit peak activity in a slightly acidic environment, thereby accelerating the degradation of the ECM (135). In OA, the slowing of ECM synthesis may also be attributable to reduced levels of glutamine—a key component in collagen synthesis within chondrocytes (143).

### 5.2 Macrophage activation and immune responses lead to ECM degradation

It is well known that, as with all inflammatory responses, a robust immune reaction occurs within the cartilage tissue of OA. Among these, cells mediating inflammation and immune defense functions are termed M1 macrophages. Furthermore, they mediate chondrocyte injury and degradation of the cartilage ECM, thereby exacerbating the progression of OA (144). Conversely, M2 macrophages mediate immune repair and anti-inflammatory functions. It is worth noting that macrophages are not typically resident in healthy articular cartilage tissue; rather, they are primarily distributed in periarticular structures such as the synovial tissue, synovial fluid, and infrapatellar fat pad during inflammatory states (145, 146). As OA progresses, macrophages in these areas are recruited and activated, polarizing into a pro-inflammatory phenotype, releasing large amounts of pro-inflammatory cytokines (such as TNF-α, IL-1β, and IL-6) and matrix-degrading enzymes (such as MMP-13 and ADAMTS-5) (147). Through paracrine effects, they disrupt chondrocyte homeostasis, promote ECM degradation, and exacerbate synovial inflammation and bone remodeling, ultimately leading to cartilage degeneration (145, 146).

Research indicates that mechanical loading may promote OA via the PIEZO1-mesenchymal stem cell-HK II-macrophage migration inhibitory factor - macrophage axis (100). Removing macrophages from cell suspensions derived from synovial samples of OA patients reduces MMP levels in the suspension (148). In preclinical OA models, intra-articular injection of chloropyrrolidone-liposome complexes to eliminate macrophages significantly reduces MMP-mediated ECM degradation (149). Conversely, M1 macrophages activated by inflammasomes such as NLRP3 release inflammatory mediators including TNF, IL-1α, IL-6, and IL-8, thereby stimulating chondrocytes to produce ROS and enhancing MMP-mediated ECM degradation (144, 150, 151).

## 6 Discussion

OA has long been regarded as a degenerative disease driven by mechanical wear and tear, however, in recent years, with the advancement of multi-omics technologies, a growing body of evidence suggests that metabolic and inflammatory dysregulation—alongside mechanical factors, may be a core driving force in OA. Currently, the academic community’s understanding of the pathological nature of OA is shifting from “local joint wear” to “systemic metabolic dysfunction.” Previously, scholars have proposed classifying OA based on metabolic types rather than traditional clinical classifications. However, it must be acknowledged that this classification currently relies primarily on basic research and preclinical models and has not yet been widely validated in real-world clinical practice or established as standardized diagnostic criteria. At this stage, the direct application of metabolic subtypes in clinical settings still lacks support from large-scale clinical studies. This review suggests that future research on chondrocyte metabolism in OA will primarily follow two major directions: the first is to integrate multi-omics studies to advance the concept of metabolism-driven OA toward clinical application; the second is to explore the mechanisms of metabolic reprogramming.

### 6.1 Research on clinical precision treatment for OA

While there have been numerous clinical studies on OA to date, the concepts and focus of previous clinical research urgently need to shift in light of the concept of precision treatment for OA based on metabolic subtypes.

#### 6.1.1 Metabolic subtype-based basket trial and omics analysis

Previous clinical studies have primarily grouped patients with OA using methods such as the KL classification; In the future, basket trials should be conducted based on patients’ baseline cartilage metabolic status (as determined by plasma or synovial fluid test results), assigning patients with the same metabolic subtype—rather than the same traditional clinical classification—to the same targeted therapy group. This approach, combined with multi-time-point metabolomics, proteomics, and transcriptomics analyses, is expected to help clarify the dynamic evolution patterns of each metabolic subtype and may provide evidence for the efficacy of precision therapy.

#### 6.1.2 Development of biomarkers and clinical predictive models using artificial intelligence

In the future, it will be necessary to actively identify stable biomarkers for OA that target the core metabolic changes in osteoarthritic chondrocytes we have proposed: impaired OXPHOS, increased glycolysis, and increased amino acid catabolism. Potential future metabolic biomarkers include:

Impaired OXPHOS: Potential clinically measurable indicators include free mitochondrial DNA released into synovial fluid due to mitochondrial damage, abnormal accumulation of intermediate products in the tricarboxylic acid cycle, and markers of oxidative stress.

Increased glycolysis: Future diagnostic criteria may rely on significantly elevated lactate concentrations in synovial fluid, as well as abnormal expression of specific rate-limiting enzymes of glycolysis in blood or synovial fluid.

Increased amino acid catabolism: Attention should be focused on changes in the amino acid metabolic profile in synovial fluid or blood, such as an imbalance in the glutamate/glutamine ratio.

Previous studies have shown that traditional single biomarkers often yield poor results. Currently, with advances in proteomics and metabolomics technologies—and potentially through the use of artificial intelligence—integrating DR, clinical indicators, and multi-omics data to construct non-invasive predictive models capable of forecasting disease progression may prove more robust than relying on a single biomarker. Such models could also be used for precision diagnosis and treatment, generating personalized “metabolic profiles” and treatment roadmaps for each OA patient.

Furthermore, efforts should be made to explore the integration of personalized “metabolic profiles” into stepwise treatment pathways: for low-risk patients, lifestyle interventions and routine follow-up should be maintained; for medium- and high-risk patients, precision drug or biologic therapy strategies guided by omics data should be initiated.

Ultimately, by developing clinical practice guidelines, we should clarify the indications, implementation protocols, and outcome interpretation standards for multi-omics + AI models in OA management, and conduct health economic evaluations to validate their incremental value in improving clinical outcomes and reducing long-term healthcare costs. Only by completing the development and validation of the aforementioned standardized processes can metabolism-subtype-driven OA treatment truly transition from a research tool to an integral part of routine clinical decision-making, thereby achieving a precision medicine transformation in OA—moving from a “one-size-fits-all” approach to a “tailored” one.

To provide a concise overview of the currently available small-molecule drugs that target different metabolic pathways and improve energy metabolism in chondrocytes, we have included **Table 3** to clearly illustrate the various drugs and their sites of action.

**Table 3:** Metabolic therapeutic agents for OA in existing literature.

Pathway	Target	Drug	Research model	References
Glycolytic Pathway	PEN/AMPK	Metformin	DMM Mice, ACLT Mice	(152–154)
	NFκB	Chrysin	Human OA Chondrocytes	(155)
	PFKFB3	Songorine	ACLT Rat	(13, 156)
	HIF-1α	α-Solanine	Rat	(121)
	LDHA	Oxamate	ACLT Rat	(120)
Mitochondrial Pathway	SIRT1/PGC-1α	Evodiamine	DMM Mice, ACLT Rat	(46, 157, 158)
	Nrf2	Curcumin	DMM Mice, MIA Rat, Human	(75, 159)
	Nrf2	Quercetin	DMM Rat, MIA Rat	(160–162)
	Nrf2	Mitoquinone	DMM Mice	(163)
	Bnip3	Tiopronin	DMM Mice	(45)
Glutamine Pathway	JNK	L-glutamine	DMM Mice	(130)

This table lists small-molecule drugs (excluding macromolecular therapies such as miRNAs) that target chondrocyte energy metabolism, as reported in recent clinical or preclinical literature, along with their targets.

### 6.2 Exploration of metabolic reprogramming mechanisms

Presently, substantial gaps and contradictions remain in understanding the mechanisms underlying chondrocyte metabolic reprogramming and subsequent responses. For instance, while most existing literature indicates that activating the SIRT1 pathway improves mitochondrial function and thereby alleviates OA, other studies suggest that SIRT1 pathway activation depletes mitochondrial NAD^+^, reducing mitochondrial ATP production and thereby promoting inflammation. The authors hypothesize that this may be related to the timing and intensity of SIRT1 activation. Early SIRT1 activation may enhance OXPHOS efficiency; however, excessive SIRT1 activation could lead to NAD^+^ pool depletion, inhibiting OXPHOS. Numerous similar issues amply demonstrate that OA arises not from alterations in a single pathway, but from a complex network of multiple pathway changes. Metabolomic time-series analysis remains necessary to elucidate the mechanisms.

Furthermore, recent studies have revealed the important role of the “gut microbiota–gut FXR–GLP-1–articular cartilage axis” in OA (164), providing strong evidence that extra-articular organs regulate OA through hormones, signaling molecules, and other mechanisms. However, the mechanism underlying the “bidirectional dialogue” between the microbiota and host chondrocytes has not yet been fully established. Current evidence primarily supports a unidirectional influence (gut → joint) (165, 166); however, whether local joint inflammation or metabolites can modulate gut microbiota composition via neuroendocrine or immune signaling feedback to form a positive feedback loop in the “gut-joint axis” requires further investigation. Furthermore, current data are limited regarding whether different microbial metabolites exhibit cross-reactions or synergistic/antagonistic effects in regulating cartilage metabolism. Resolving these questions relies heavily on integrated multi-omics analyses. Future research should focus on the following areas: (1) Longitudinal cohort and intervention studies to validate causal relationships, such as using fecal microbiota transplantation, probiotic interventions, or FXR/GLP-1 agonists, to evaluate their impact on OA progression; (2) Establishing a bidirectional axis model using gene-knockout mice or joint-gut co-culture systems to investigate the feedback regulation of gut microbiota composition by joint inflammatory signals; (3) Conducting multi-omics integrated analyses of cross-reactions among specific microbial metabolites to develop precision intervention strategies targeting the microbiome.

Additionally, there is a significant research gap regarding the interaction between metabolic reprogramming and epigenetic modifications. As mentioned earlier, chondrocytes undergo metabolic reprogramming—such as enhanced glycolysis and impaired OXPHOS—during the progression of OA, accompanied by epigenetic modifications like histone lactylation. However, most studies still treat metabolic reprogramming and epigenetic changes as isolated events or simple upstream-downstream relationships, overlooking the complex positive feedback loops they may form. For example, lactate is not only an increased end product of metabolic reprogramming in OA but also a key substrate for histone lactylation. In OA chondrocytes, enhanced glycolysis leads to lactate accumulation, which may further promote glycolysis, inflammation, and cell death by inducing lactylation modifications of key genes, thereby forming a vicious “metabolic-epigenetic-inflammatory-metabolic” positive feedback loop (109). Future research directions include: (1) Constructing dynamic multi-omics models to elucidate the roles of different lactylation sites at various stages of OA, as well as the quantitative dynamics of the positive feedback loop; (2) Developing specific intervention tools to validate the efficacy of disrupting the “metabolic-epigenetic-inflammatory” loop in protecting cartilage and to assess the potential for synergy with existing therapies; (3) Clinical translation studies, including the detection of lactylation markers in synovial fluid from OA patients as biomarkers, or the development of nanodelivery systems targeting lactylation metabolism to enable precision therapy.

### 6.3 Integrating biomechanics into the metabolic framework of OA

**Figure 2:**
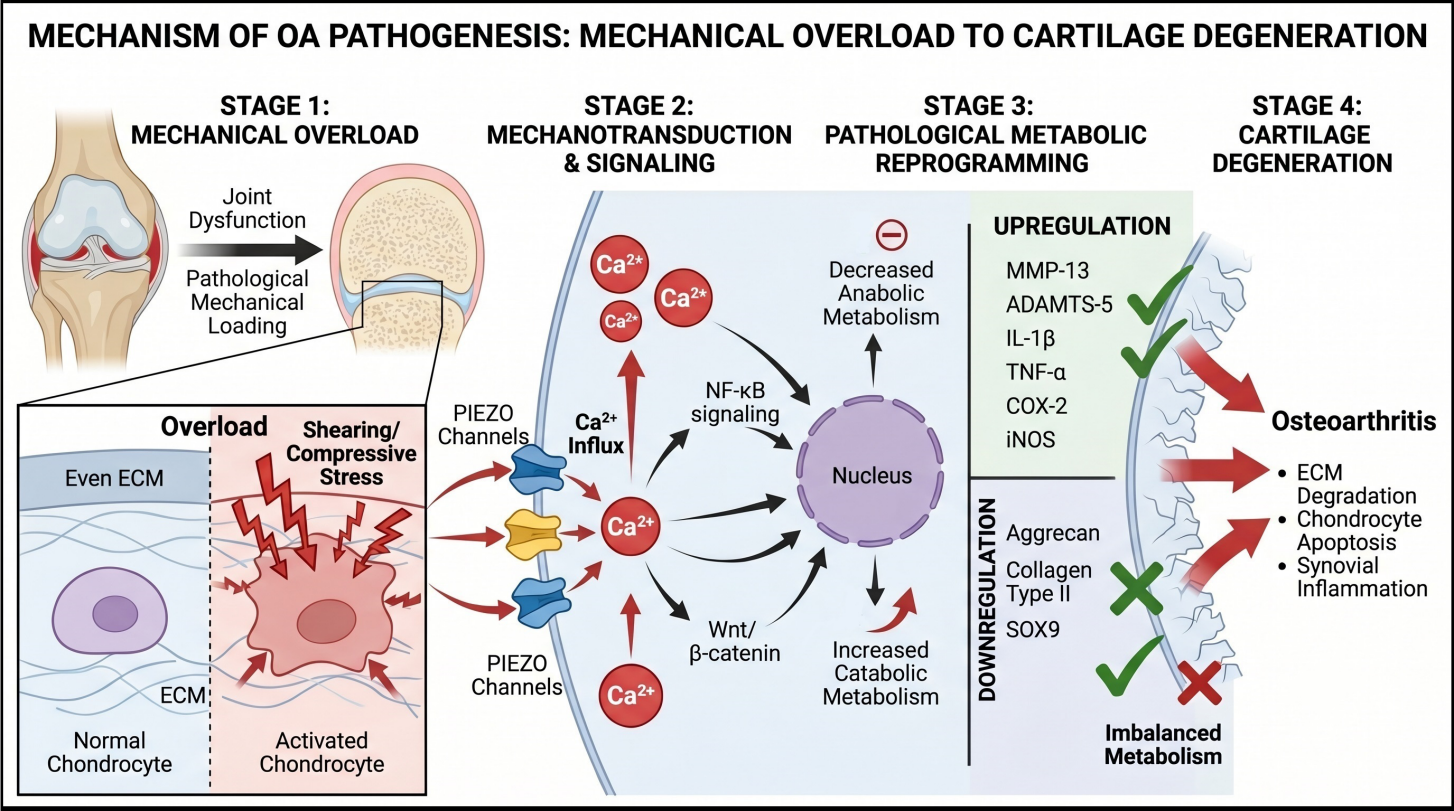
Diagram of mechanical overload mechanism. This image illustrates that joint dysfunction often leads to pathological mechanical overload, which activates mechanoreceptors on chondrocytes (such as PIEZO), thereby directly triggering pathological metabolic reprogramming and ultimately leading to OA.

While this review focuses on metabolic reprogramming, it is crucial to recognize that this does not occur in a vacuum. A comprehensive disease model must position biomechanical overload as a upstream driver of the metabolic energy crisis. Pathological loading activates mechanoreceptors (e.g., PIEZO channels) on chondrocytes, which directly trigger the metabolic hallmarks discussed above (**Figure 2**). Future research should aim to dissect the crosstalk between biomechanical overload and metabolic reprogramming, by integrating multi-omics analyses of mechanically stimulated chondrocytes and developing computational models that link joint-level biomechanics with cellular-level metabolism. Furthermore, a comprehensive therapeutic approach for OA will likely require a combination of metabolic interventions (e.g., targeting glycolysis or mitochondrial dysfunction) with biomechanical strategies (e.g., joint offloading or gait modification) to effectively break the vicious cycle of mechano-induced metabolic collapse.
